# The Clinical Efficacy of Yindanxinnaotong Soft Capsule in the Treatment of Stroke and Angina Pectoris: A Meta-Analysis

**DOI:** 10.1155/2017/2060549

**Published:** 2017-04-30

**Authors:** Long Cheng, Yue Liu, Xiao-bo Sun

**Affiliations:** ^1^Key Laboratory of Bioactive Substances and Resources Utilization of Chinese Herbal Medicine, Ministry of Education, Institute of Medicinal Plant Development, Chinese Academy of Medical Sciences and Peking Union Medical College, Beijing 100193, China; ^2^Xiyuan Hospital, China Academy of Chinese Medical Sciences, Beijing 100053, China

## Abstract

*Objective*. To systematically evaluate the clinical efficacy of Yindanxinnaotong (YD) soft capsule in adult patients with cardiovascular diseases (stroke and angina pectoris).* Methods*. We electronically searched databases including Medline, PubMed, Chinese National Knowledge Infrastructure (CNKI), Cqvip Database (VIP), and Wanfang Database for published articles of randomized controlled trials (RCTs) of YD capsule in treating stroke and angina pectoris. The meta-analysis was performed using RevMan 5.3 software.* Results*. 49 RCTs involving 6195 subjects with cardiovascular diseases (angina pectoris and stroke) were included. Compared with western conventional medicine (WCM) and/or other Chinese medicines, YD plus WCM therapeutic regimen could significantly improve the efficacy rate (RR = 1.21, 95% CI (1.17, 1.25), *P* < 0.00001 for angina pectoris, RR = 1.24, 95% CI (1.18, 1.31), *P* < 0.00001 for stroke), showing the clinical value. In addition, the therapeutic efficiency of WCM plus YD capsule regimen is better than that of WCM alone in improving CRP (MD = −2.07, 95% CI (−3.97, −0.17), *P* = 0.03 <0.05) and TG (MD = −0.37, 95% CI (−0.52, −0.23), *P* < 0.0001).* Conclusion*. YD is effective in the treatment of cardiovascular diseases (angina pectoris and stroke) in adults, and WCM plus YD therapeutic regimen can significantly improve the effective rate in the clinic.

## 1. Introduction

Stroke and coronary heart disease (CHD) are the leading causes of morbidity, mortality, and health care expenditure in adults especially the elderly. Both diseases share risk factors and pathogenic processes, that is, atherosclerosis and thrombosis. Even if the most advanced, comprehensive treatment is applied, still more than 50% of the survivors from cerebrovascular accidents cannot live completely and take care of themselves [1, 2]. Stroke and angina pectoris have the characteristics of high incidence, high mortality, high morbidity, high recurrence rate, and complications. At present, China has more than 270 million patients with stroke and angina pectoris. Nearly 3 million patients with stroke and angina pectoris died in China every year, accounting for 51% of China's annual total death [3].

Yindanxinnaotong (YD) capsule is a Chinese patent drug processed by modern advanced pharmaceutical technology with improved formulations, which refined the characteristics of traditional Miao ethnic medicine. The formulations of YD include ginkgo leaves, salvia miltiorrhiza, herba erigeromtis,* Gynostemma pentaphyllum*, hawthorn, allium sativum,* Panax notoginseng*, and borneol [4]. YD has the function of promoting blood circulation, removing blood stasis, and relieving pain [3]. YD has the indications of Qi stagnation and blood stasis caused by obstruction of Qi in the chest; and treatment includes chest pain, chest tightness, shortness of breath, and heart palpitations and angina pectoris of coronary heart disease, hyperlipidemia, cerebral arteriosclerosis, stroke, stroke sequelae, and so forth. Modern pharmacological studies have shown that YD can expand coronary artery, increase the coronary blood flow, smooth vascular muscle, reduce myocardial consumption oxygen, prevent and treat myocardial ischemia, reduce platelet aggregation and blood lipids, and protect from the ischemia reperfusion injury [5, 6].

In China, Chinese medicines play an irreplaceable role in the prevention and treatment of cardiovascular diseases, and more and more doctors tend to apply the combined regimen (western conventional medicine (WCM) plus Chinese medicine), to improve cardiovascular disease treatment outcome [7–10]. However, the safety and effectiveness of Traditional Chinese Medicine (TCM) in clinical applications are not unified and need more scientific and standardized assessment data to provide reliable evidence for its clinical practice [11, 12]. Given present situation, the research aims to evaluate the appropriate use of YD capsule in treatment of stroke and angina pectoris through meta-analysis and literature research methods and promote the rational use of YD. Researches have confirmed that the high sensitive C-reactive protein (hs-CRP) and triglyceride (TG) are closely related to the development of atherosclerosis. In this study, we also comprehensively evaluated the effect of Yindanxinnaotong capsule on the above indexes.

## 2. Material and Methods

### 2.1. Inclusion and Exclusion Criteria

Studies of randomized controlled trials were included, the published language is not limited, regardless of whether the study was blinded in grouping. The specific inclusion criteria were the following.

1. Subjects were included and diagnosed as stroke or angina pectoris.
2. The treatment group received western conventional medicine (WCM, Table 1) plus YD capsule treatment; the control group received WCM alone.
3. All included research literature must be able to extract one of the following results: the primary outcome was angina pectoris symptoms, clinical efficacy, or nerve function defect score; the secondary outcome measures included electrocardiogram, angina attack frequency and duration, blood rheology, and lipids characters.

The exclusion criteria were the following.

1. The included literatures were nonrandomized, controlled trials.
2. The studies being animal experiments or clinical pharmacokinetic studies, or the study enrolling healthy subjects.
3. The research data has obviously large deviation.
4. The design scheme of the research is not clear, or the data is not complete.

### 2.2. Interventions

The interventions regimen was WCM plus YD treatment or WCM treatment alone. (1) WCM treatment of coronary heart disease and angina pectoris includes nitrates, aspirin, and amlodipine; (2) WCM treatment for stroke includes nimodipine, flunarizine, and aspirin; (3) angina pectoris routine treatment includes nitrous esters, aspirin, amlodipine, and isosorbide dinitrate; (4) the treatment dose of YD capsule was referenced according to drug directions; the courses ranged from 2 to 12 weeks.

The final outcome/expected outcome criteria should clearly include at least one of the following outcomes: (1) at the end of treatment or follow-up period, clinical symptoms ease, ECG efficacy or other clinical symptoms of improvement, or the total clinical efficiency rate was expressed; (2) the therapeutic effect was compared as blood lipids (total cholesterol, glycerin trilaurate, low density lipoprotein cholesterol, and high density lipoprotein cholesterol) changes at the end of treatment or at follow-up end; (3) score of blood rheology index changes exist (plasma specific viscosity, fibrinogen, and C-reactive protein); (4) the change of Barthel index score, neurological deficit score, and life ability score exist in stroke at the end of treatment or follow-up.

*Literature Source.* The literatures were collected through computer retrieval system, the database including PubMed, Medline, China Biology Medicine disc (CBMDisc), Chinese Medical Academic Conference papers database (CMAC), China National Knowledge Infrastructure (CNKI), Chinese science and technology journal full-text database (VIP), Web of Science, and Wanfang Database from 2007 to 2016; the retrieval deadline is June 2016. And all literatures that met the inclusion criteria of references and are relevant to YD capsule in the cardiovascular prognosis were searched one by one for data missing.

*Retrieval Method.* We screened above retrieval database with the key words of “YD capsule”, “Yindanxinnaotong capsule”, and YDXNT capsule; “angina pectoris” and “stroke”. The language was not restricted; limited conditions include “human” and “random of test”. We also use Google Scholar, Baidu scholar, and other search engines and manual retrieval as a supplement to trace included references. Retrieved literatures are imported into duplicate check software in order to remove duplication and manage document.

*Literature Selection and Data Extraction.* There were 2 reviewers independently screening and cross-checking these literatures according to predefined inclusion criteria. Disagreement was addressed to a third researcher. The lack of information was solved through contacting the original author. Through screening the title, abstract, and text, reviewers determined whether the final data could be included. The mainly included contents were basic information of the study, including research title, first author, published journal, and time; the key elements of study design and quality evaluation; the basic situation of experimental group and the control group, including the number of cases included and age; the effectiveness, safety, and so on.

*Bias Risk Assessment.* The bias risk assessment was performed by 2 reviewers in accordance with the Cochrane collaboration group for randomized controlled trials by tools [13, 14]. The evaluated contents included (1) random allocation method; (2) allocation of groups; (3) the blind method applied in research subjects and researchers; (4) the results of data integrity: including baseline measurements before the intervention and effect parameters after intervention, drop out/exit (whether the dropout rate is less than 10%), and whether the loss reason was explained whether drop out/existing patients' data was analyzed by the intent to treat (ITT) analysis; (5) the selective reporting results: for the negative result, whether the security issues (death and other adverse events) were reported; (6) other sources of bias including early stop of test and baseline imbalance. “Yes” in point (6) means the bias was low, “no” means high bias, and “unclear” means the lack of relevant information or bias uncertainty.

*Data Analysis.* The RevMan 5.3 software was used for statistical analysis. The binary classification data were expressed as odds ratio (OR) and 95% CI. The continuous data were expressed as mean difference (MD) and 95% CI. Chi square test (alpha = 0.1) was used for heterogeneity analysis and the heterogeneity of size was quantitatively assessed combined with *I*^2^. If *P* > 0.05, *I*^2^ ≤ 50%, fixed effects model was used; if there is heterogeneity, the sources of heterogeneity should be examined and removed; if the heterogeneity still exists, and the study has clinical homogeneity, meta-analysis was performed using random effect model. If the heterogeneity could not be combined, the descriptive analysis was adopted.

## 3. Result

**Figure 1:**
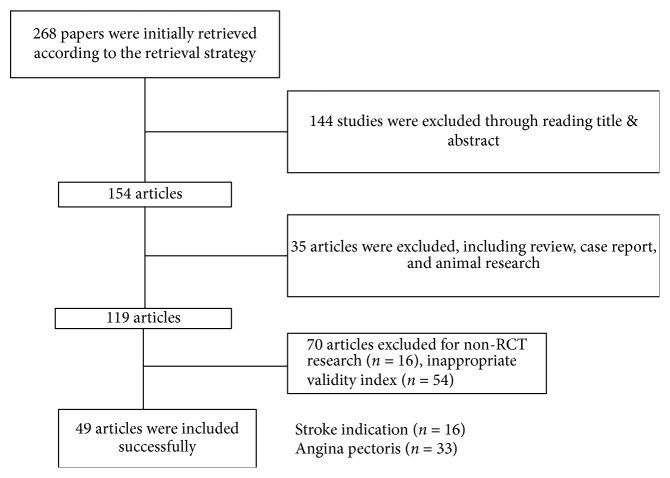
The literature screening result.

According to retrieval strategy, a total of 268 related literatures were retrieved, through reading the title and abstract, and 114 articles were excluded for not meeting the inclusion criteria and involving animal study. 35 articles containing duplicated research, nonrandomized control study, interventions, and end index which does not meet inclusion criteria were removed; then, the other 5 literatures with clear deviation of outcome indicators were removed, and 119 articles were included for further review. The final included RCTs were 49 (Figure 1).

### 3.1. The Characteristics of Included Study and the Risk Assessment of Bias

The 49 RCT studies [15–63] included 6195 patients with stroke and angina pectoris. The minimum sample size of each group was 22, the maximum sample size was 201. The basic characteristics of the included studies are shown in Table 1, and the bias risk assessment results are shown in Table 2.

### 3.2. Stroke Curative Effect Analysis

**Figure 2:**
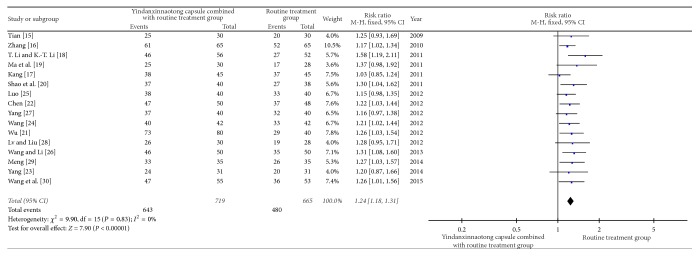
Meta-analysis of therapeutic effect of YD on stroke.

**Figure 3:**
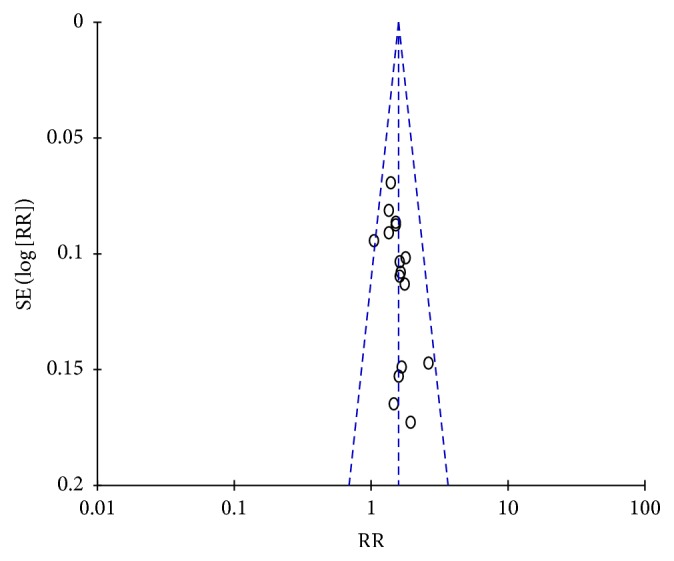
Funnel plot of the therapeutic effect of YD on stroke.

A total of 1384 patients in 16 random control trial (RCT) [15–30] were included. The fixed effect model in meta-analysis showed that the therapeutic effect of WCM plus YD capsule (in 719 patients) regimen is better than WCM alone regimen treatment (in 665 patients) (RR = 1.24, 95% CI: 1.18, 1.31; *P* < 0.00001) (Figure 2). Funnel plot (Figure 3) showed that the literature is symmetrical, with Eggers test of *P* > 0.05. There is no publication bias. Results showed that YD capsule combined with conventional treatment can control and improve the clinical symptoms of stroke, indicating obvious clinical value.

### 3.3. Curative Effect of YD on Angina Pectoris

**Figure 4:**
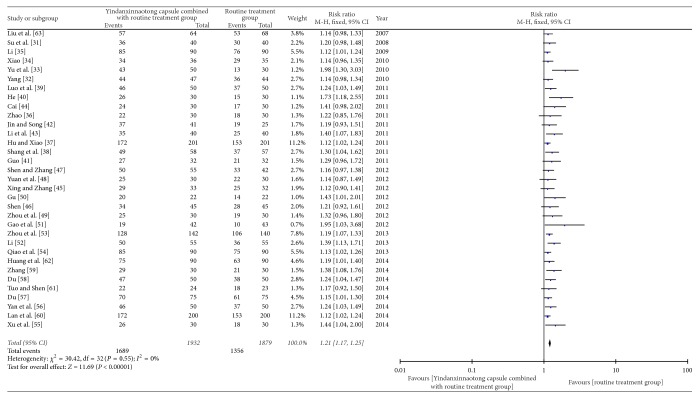
Meta-analysis of therapeutic effect of YD on angina pectoris.

**Figure 5:**
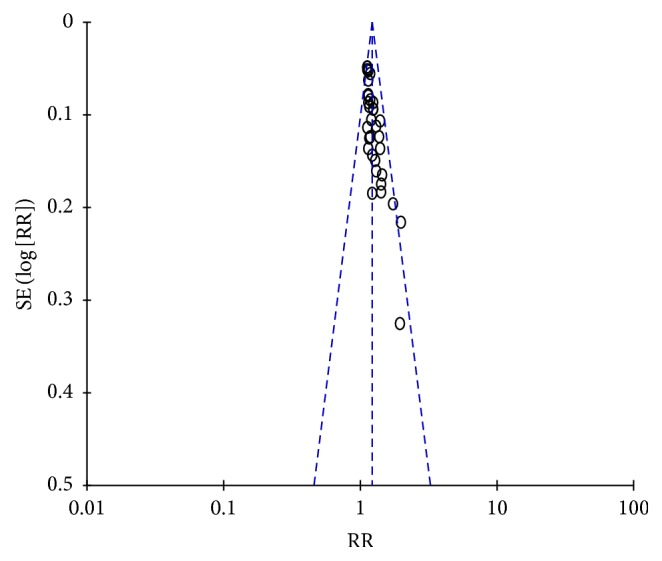
Funnel plot of the therapeutic effect of YD on angina pectoris.

3811 patients in 33 RCTs [31–63] were included: 1932 received WCM plus YD capsule and 1879 received WCM. The fixed effect model in meta-analysis showed that the therapeutic efficiency of WCM plus YD capsule regimen is better than that of WCM alone regimen treatment (RR = 1.21, 95% CI: 1.17, 1.25; *P* < 0.00001) (Figure 4). The funnel plot (Figure 5) showed that the literatures were distributed centrally and symmetrically. The Eggers test result was *P* > 0.05; there is no publication bias.

### 3.4. Effect of YD on CRP and TG

**Figure 6:**
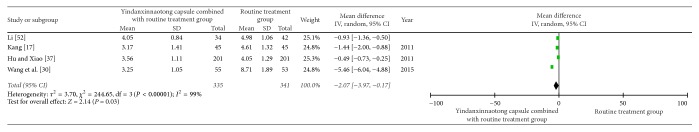
Meta-analysis of therapeutic effect of YD on CRP.

**Figure 7:**
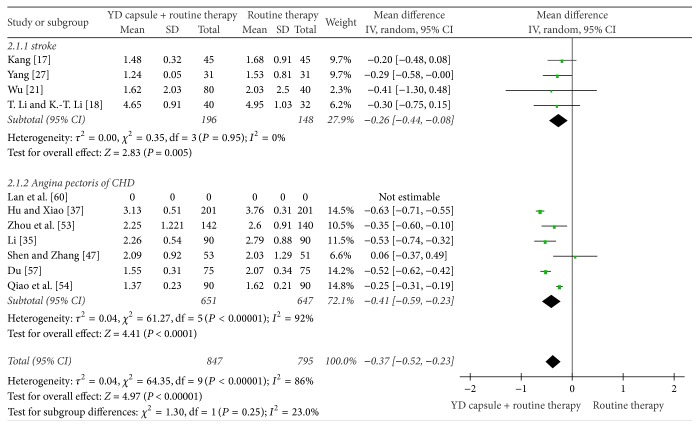
Meta-analysis of therapeutic effect of YD on TG.

The fixed effect model in meta-analysis showed that the therapeutic efficiency of WCM plus YD capsule regimen is better than that of WCM alone in improving CRP [17, 30, 37, 52]: MD = −2.07, 95% CI (−3.97, −0.1), *P* = 0.03, Figure 6 and TG [17, 18, 21, 27, 35, 47, 54, 57, 60]: MD = −0.37, 95% CI (−0.52, −0.23), *P* < 0.0001, Figure 7.

In addition, analysis results also demonstrated that YD could regulate coagulation condition and fibrinolytic system in patients with coronary heart disease and improve myocardial ischemia, angina symptoms, and heart function. Administration of YD reduced restenosis after interventional treatment. YD can not only treat coronary heart disease, but also regulate blood lipids, which are important risk factor for coronary heart disease. Administration of YD could control the long-term development of the cardiovascular disease and prevent the cardiovascular events from happening again; YD has important significance for patients with cardiovascular disease.

## 4. Discussions

Prior to the development of modern pharmaceutical drugs, doctors of Traditional Chinese Medicine (TCM) treated angina and other symptoms of coronary artery disease through the use of acupuncture, herbal remedies, qigong, and tai chi. Today, Chinese Traditional Medicine therapies are proving to still be useful for cardiovascular disorders. Although patients may be diagnosed with different diseases according to western medicine, if the cause of the disease is the same according to TCM diagnosis and differentiation, their treatment principle will be the same.

Chinese medicine believes that cardiovascular disease is categorized to “heartache, palpitations, chest pain” in Traditional Chinese Medicine theory; and the cerebrovascular diseases could be collectively referred to as “stroke”; the weakness of blood circulation that leads to blood stasis is the important reason for the formation of cardiovascular disease. “Deficiency” is the fundamental reason of the 2 diseases and “stasis” is the direct reason. The key treatment in cardiovascular disease is to invigorate Qi, promote blood circulation, and remove blood stasis. The western medicine theory categorized lesions occurring in the cardiac artery as cardiovascular disease, like coronary heart disease, angina pectoris, myocardial infarction, and so on. Cerebral stroke, also known as the “stroke,” is a group diseases of brain vessel injury due to brain vessel rupture or vascular flow obstruction. According to the change of clinical manifestations, stroke can be divided into two categories: hemorrhagic and ischemic stroke, which include commonly occurring cerebral embolism and cerebral hemorrhage in clinic [64–66]. The main pathology of the 2 diseases is atherosclerosis; the major complication of atherosclerosis is thrombosis, which could cause local arterial/distal embolization and lead to various vascular diseases [67, 68]. The standardized treatment in western medicine is mainly to deal with cardiovascular events, including expanding coronary artery, controlling blood pressure, reducing blood lipid and blood viscosity, and anticoagulation and antiplatelet aggregation treatment.

In this paper, RCT literatures on the efficacy of YD and commonly used WCM or Chinese medicine in clinical treatment of stroke and angina pectoris have been systematically evaluated and analyzed. Results indicate that YD capsule combined with routine treatment has significantly higher efficiency than conventional treatment in improving clinical symptoms of patients with stroke or angina pectoris and improves the cardiovascular related laboratory index, such as ECG, decreasing blood lipid. This might be because the YD contains multibioactive substances [69, 70]; Ginkgolides can scavenge oxygen free radicals and improve microcirculation and myocardial hypoxia [67]; Gynostemma has antiplatelet and inhibits thrombosis and blood lipid lowering effect [71]. So YD can improve the blood flow, increase cardiovascular support, repair vascular elasticity, reduce blood viscosity, keep blood flowing, and regulate blood pressure auxiliary. YD could be comprehensively used in different stages of cardiovascular disease. It is especially suited for patients with stroke and angina pectoris simultaneously; it could improve the associated symptoms caused by cardiovascular disease and regulate the blood lipid conditioning and prevent the occurrence of cardiovascular event.

In summary, for patients with stroke and angina pectoris, YD capsule combined with routine medication has certain advantages in improving clinical symptoms and laboratory indexes. Due to lack of long-term follow-up data, long-term efficacy and safety of YD can not be assessed. Therefore, the confirmative conclusions are not allowed. High-quality, large sample, multicenter randomized clinical trials still need to be done in the future.

## Figures and Tables

**Table 1:** The basic characteristics of final included studies.

Author	Indication	Male	Age		Case number		Intervention methods		Outcome measurement	Courses
			Test group	Controls	Test group	Controls	Test group	Controls		
Tian [15]	Cerebral arterial thrombosis	21/19	56.45 ± 9.03	55.40 ± 10.48	30	30	4 pills of YD capsule, tid + conventional treatment	Conventional therapy (flunarizine, nimodipine, hydroxyethylrutin, etc.)	① ②③⑤	45 d
Zhang [16]	Cerebral arterial thrombosis	45/42	39~81	37~84	65	65	4 pills of YD capsule, tid + conventional treatment	Conventional antipressure & cerebral protection therapy	① ②	28 d
Kang [17]	Cerebral infarction	24/25	45~75	47~75	45	45	4 pills of YD capsule, tid + conventional treatment	Conventional therapy (atorvastatin, aspirin)	① ④	84 d
T. Li and K.-T. Li [18]	Cerebral infarction	31/29	37~75	35~75	56	52	4 pills of YD capsule, tid + conventional treatment	Conventional therapy (aspirin)	① ②③	14 d
Ma et al. [19]	Cerebral infarction	18/18	55~72	58~74	30	28	4 pills of YD capsule, tid + conventional treatment	Conventional antiplatelet & antilipid therapy	① ②③	28 d
Shao et al. [20]	Cerebral infarction	25/22	48~78	45~75	40	40	4 pills of YD capsule, tid + conventional treatment	Conventional antiplatelet & antilipid therapy	① ②⑤	56 d
Wu [21]	Cerebral infarction	49/22	NA	NA	80	40	4 pills of YD capsule, tid + conventional treatment	Conventional therapy (mannitol, etc.)	① ③④	28 d
Chen [22]	Cerebral infarction	25/22	48~78	47~75	50	48	4 pills of YD capsule, tid + conventional treatment	Conventional antiplatelet & antilipid therapy	① ②⑤	56 d
Yang [23]	Cerebral arterial thrombosis	NA	NA	NA	40	40	4 pills of YD capsule, tid + conventional treatment	Conventional therapy (mannitol, etc.)	① ②⑤	21 d
Wang [24]	Cerebral arterial thrombosis	NA	NA	NA	42	42	4 pills of YD capsule, tid + conventional treatment	Conventional therapy	①	NA
Luo [25]	Cerebral arterial thrombosis	24/40	55~85	NA	40	40	4 pills of YD capsule, tid + conventional treatment	Conventional therapy (nimodipine, etc.)	① ②⑤	30 d
Wang and Li [26]	Cerebral arterial thrombosis	30/28	61.8 ± 3.2	61.3 ± 3.4	50	50	4 pills of YD capsule, tid + conventional treatment	Conventional therapy	①	NA
Yang [27]	Cerebral infarction	20/19	44~77	41~74	31	31	4 pills of YD capsule, tid + conventional treatment	Conventional therapy (aspirin, etc.)	① ③④	30 d
Lv and Liu [28]	Cerebral apoplexy	17/15	61.4 ± 5.3	62.7 ± 4.5	30	28	4 pills of YD capsule, tid + conventional treatment	Conventional therapy (nimodipine, etc.)	① ②③④⑤	30 d
Meng [29]	Cerebral infarction	17/19	48~80	50~83	35	35	4 pills of YD capsule, tid + conventional treatment	Conventional therapy	① ③	14 d
Wang et al. [30]	Cerebral infarction	25/26	62 ± 3.8	58 ± 4.3	55	53	4 pills of YD capsule, tid + conventional treatment	Conventional therapy (aspirin, etc.)	① ③	90 d
Su et al. [31]	Angina pectoris, CHD	34/32	60~80	60~79	40	40	4 pills of YD capsule, tid + conventional treatment	Conventional therapy (nitrates, aspirin, etc.)	① ⑥	84 d
Yang [32]	Angina pectoris, CHD	28/22	49~73	47~69	47	46	4 pills of YD capsule, tid + conventional treatment	Conventional therapy (nitrates, aspirin, etc.)	① ⑥	30 d
Yu et al. [33]	Angina pectoris	NA	NA	NA	50	30	4 pills of YD capsule, tid + conventional treatment	Conventional therapy (nitrates, aspirin, etc.)	① ④⑥⑦	28 d
Xiao [34]	Angina pectoris, CHD	22/22	60~74	60~71	36	35	4 pills of YD capsule, tid + conventional treatment	Conventional therapy (nitrates, *β*-receptor blocker, aspirin, etc.)	① ⑥	56 d
Li [35]	Angina pectoris, CHD	NA	NA	NA	45	45	4 pills of YD capsule, tid + conventional treatment	Conventional therapy (aspirin, calcium antagonist, etc.)	① ④⑥⑦	56 d
Zhao [36]	Angina pectoris, CHD	18/16	45~84	44~79	30	30	4 pills of YD capsule, tid + conventional treatment	Aspirin and nitric acid	① ⑥	30 d
Hu and Xiao [37]	Angina pectoris	108/112	38~91	40~89	201	201	4 pills of YD capsule, tid + conventional treatment	Conventional therapy (*β*-receptor blocker, aspirin, etc.)	①	90 d
Shang et al. [38]	CHD	28/27	65.8 ± 6.1	67.1 ± 6.2	58	57	4 pills of YD capsule, tid + conventional treatment	Conventional therapy (nitrates, *β*-receptor blocker, aspirin, etc.)	① ④	56 d
Luo et al. [39]	Angina pectoris, CHD	34/32	48~73	50~72	50	50	4 pills of YD capsule, tid + conventional treatment	Conventional therapy (*β*-receptor blocker, calcium antagonist, aspirin, etc.)	① ④⑥	56 d
He [40]	Angina pectoris, CHD	17/18	43~79	45~78	30	30	4 pills of YD capsule, tid + conventional treatment	Conventional therapy (nitrates, aspirin, etc.)	① ⑦	30 d
Guo [41]	Angina pectoris	18/16	81.0 ± 3.3	83.0 ± 4.4	32	32	4 pills of YD capsule, tid + conventional treatment	Conventional therapy (isosorbide mononitrate)	①⑥	28 d
Jin and Song [42]	Angina pectoris, CHD	32/20	69~78	48~79	41	25	4 pills of YD capsule, tid + conventional treatment	Conventional therapy (isosorbide mononitrate)	①⑥	30 d
Li et al. [43]	Angina pectoris, CHD	28/26	70~88	77~89	40	40	4 pills of YD capsule, tid + conventional treatment	Conventional therapy (isosorbide mononitrate)	① ⑥	30 d
Cai [44]	Angina pectoris, CHD	17/18	43~79	45~78	30	30	4 pills of YD capsule, tid + conventional treatment	Conventional therapy (nitrates, aspirin, etc.)	① ⑥	30 d
Xing and Zhang [45]	Angina pectoris, CHD	19/32	55.9 ± 13.2	56.5 ± 12.9	33	32	4 pills of YD capsule, tid + conventional treatment	Conventional therapy (nitrates, aspirin, etc.)	① ③⑥	42 d
Shen [46]	Angina pectoris, CHD	31/21	45~80	43~78	34	32	4 pills of YD capsule, tid + conventional treatment	Conventional therapy (nitrates, aspirin, etc.)	① ③	28 d
Shen and Zhang [47]	Angina pectoris, CHD	42/32	69~85	67~87	55	42	4 pills of YD capsule, tid + conventional treatment	Conventional therapy	①	84 d
Yuan et al. [48]	Angina pectoris, CHD	22/23	29~70	31~70	30	30	4 pills of YD capsule, tid + conventional treatment	Conventional therapy	① ⑥	56 d
Zhou et al. [49]	Angina pectoris	14/19	42~72	40~73	30	30	4 pills of YD capsule, tid + conventional treatment	Conventional therapy	① ③⑥	56 d
Gu [50]	Angina pectoris	15/17	55.6 ± 3.4	57.3 ± 3.6	22	22	4 pills of YD capsule, tid + conventional treatment	Conventional therapy	① ⑥	56 d
Gao et al. [51]	Angina pectoris	22/23	62~74	60~71	42	43	4 pills of YD capsule, tid + conventional treatment	Conventional therapy (nitrates, *β*-receptor blocker, hydroxyethylrutin, aspirin, etc.)	① ⑥	56 d
Li [52]	Angina pectoris, CHD	37/35	53~80	52~77	55	55	4 pills of YD capsule, tid + conventional treatment	Conventional therapy (nitrates, calcium antagonist, aspirin, etc.)	① ⑥	56 d
Zhou et al. [53]	Angina pectoris, CHD	80/78	50~82	42~78	142	140	4 pills of YD capsule, tid + conventional treatment	Conventional therapy (isosorbide mononitrate, aspirin, etc.)	① ④⑥⑦	56 d
Qiao et al. [54]	Angina pectoris	50/42	56.8 ± 6.8	55.2 ± 5.6	90	90	4 pills of YD capsule, tid + conventional treatment	Conventional therapy	① ④	180 d
Xu et al. [55]	Angina pectoris, CHD	NA	NA	NA	30	30	4 pills of YD capsule, tid + conventional treatment	Conventional therapy (isosorbide mononitrate, aspirin, etc.)	① ⑥	56 d
Yan et al. [56]	Angina pectoris, CHD	38/36	48~69	52~71	50	50	4 pills of YD capsule, tid + conventional treatment	Conventional therapy (nitrates, *β*-receptor blocker, calcium antagonist, aspirin, etc.)	① ⑥	84 d
Du [57]	CHD	45/42	41~85	44~84	75	75	4 pills of YD capsule, tid + conventional treatment	Conventional therapy (nitrates, calcium antagonist, etc.)	① ④	56 d
Du [58]	Angina pectoris, CHD	27/24	45~84	44~79	50	50	4 pills of YD capsule, tid + conventional treatment	Conventional therapy (nitrates, *β*-receptor blocker, calcium antagonist, hydroxyethylrutin, aspirin, etc.)	① ⑥	56 d
Zhang [59]	Angina pectoris, CHD	16/17	52~78	56~83	30	30	4 pills of YD capsule, tid + conventional treatment	Conventional therapy	① ⑥	28 d
Lan et al. [60]	CHD	108/112	60~93	60~96	200	200	4 pills of YD capsule, tid + conventional treatment	Conventional therapy (nitrates, *β*-receptor blocker, calcium antagonist, aspirin, etc.)	① ④⑥	60 d
Tuo and Shen [61]	Angina pectoris	NA	NA	NA	24	23	4 pills of YD capsule, tid + conventional treatment	Conventional therapy	① ③	180 d
Huang et al. [62]	Angina pectoris, CHD	51/49	52~77	51~79	90	90	4 pills of YD capsule, tid + conventional treatment	Conventional therapy	① ④⑥	56 d
Liu et al. [63]	Angina pectoris	31/39	42~72	40~73	64	68	3 pills of YD capsule, tid + conventional treatment	Conventional therapy	① ③	56 d

*Note*. The outcome measures: ① efficiency; ② scores of nerve function defect; ③ indexes of the blood rheology; ④ the blood lipid index; ⑤ the overall score of quality of life; ⑥ efficiency of the ECG outcome; ⑦ efficacy of TCM syndrome.

**Table 2:** The risk assessment of bias for included study.

Included studies	Random methods	Allocation	Blinded methods		Results data integrity (drop out/loss up)	Selective reported results	Other bias	Jadad
			Patients and researcher	Outcome measurer				
Tian [15]	Random	NA	NA	NA	NA	NA	NA	1
Zhang [16]	Random	NA	NA	NA	NA	NA	NA	2
Kang [17]	Random	NA	NA	NA	NA	NA	NA	1
T. Li and K.-T. Li [18]	Random	NA	NA	NA	NA	NA	NA	2
Ma et al. [19]	Random	NA	NA	NA	NA	NA	NA	2
Shao et al. [20]	Random	NA	NA	NA	NA	NA	NA	2
Wu [21]	Random	Yes	Blind	NA	NA	NA	NA	3
Chen [22]	Random number tables	Yes	Blind	Yes	Yes	NA	NA	4
Yang [23]	Random number tables	NA	NA	NA	NA	NA	NA	2
Wang [24]	Random	NA	NA	NA	NA	NA	NA	2
Luo [25]	Random	NA	NA	NA	NA	NA	NA	2
Wang and Li [26]	Random	Yes	Blind	NA	NA	NA	NA	3
Yang [27]	Random	NA	NA	NA	NA	NA	NA	1
Lv and Liu [28]	Random	Yes	Blind	NA	NA	NA	NA	3
Meng [29]	Random	NA	NA	NA	NA	NA	NA	2
Wang et al. [30]	Random	Yes	Blind	NA	NA	NA	NA	3
Su et al. [31]	Random	NA	NA	NA	NA	NA	NA	1
Yang [32]	Random	Yes	Blind	NA	NA	NA	NA	3
Yu et al. [33]	Random	NA	NA	NA	NA	NA	NA	1
Xiao [34]	Random	Yes	Blind	Yes	Yes	NA	NA	4
Li [35]	Random	NA	NA	NA	NA	NA	NA	1
Zhao [36]	Random	NA	NA	NA	NA	NA	NA	2
Hu and Xiao [37]	Random	NA	NA	NA	NA	NA	NA	2
Shang et al. [38]	Random	NA	NA	NA	NA	NA	NA	1
Luo et al. [39]	Random	NA	NA	NA	NA	NA	NA	1
He [40]	Random	Yes	Blind	Yes	Yes	NA	NA	4
Guo [41]	Random	NA	NA	NA	NA	NA	NA	1
Jin and Song [42]	Random	NA	NA	NA	NA	NA	NA	2
Li et al. [43]	Random	NA	NA	NA	NA	NA	NA	1
Cai [44]	Random	NA	NA	NA	NA	NA	NA	2
Xing and Zhang [45]	Random	Yes	Double blind	NA	Yes	NA	NA	3
Shen [46]	Random	Yes	Blind	NA	NA	NA	NA	3
Shen and Zhang [47]	Random	NA	NA	NA	NA	NA	NA	1
Yuan et al. [48]	Random	Yes	Blind	NA	NA	NA	NA	1
Zhou et al. [49]	Random	NA	NA	NA	NA	NA	NA	3
Gu [50]	Random	NA	NA	NA	NA	NA	NA	1
Gao et al. [51]	Random	NA	Blind	NA	NA	NA	NA	3
Li [52]	Random	NA	Blind	NA	NA	NA	NA	3
Zhou et al. [53]	Random	NA	NA	NA	NA	NA	NA	1
Qiao et al. [54]	Random	Yes	Blind	Yes	Yes	NA	NA	4
Xu et al. [55]	Random number tables	NA	NA	NA	NA	NA	NA	2
Yan et al. [56]	Random	NA	NA	NA	NA	NA	NA	1
Du [57]	Random number tables	Yes	Blind	NA	NA	NA	NA	3
Du [58]	Random number tables	Yes	Blind	NA	NA	NA	NA	3
Zhang [59]	Random	NA	NA	NA	NA	NA	NA	1
Lan et al. [60]	Random	NA	NA	NA	NA	NA	NA	2
Tuo and Shen [61]	Random	NA	NA	NA	NA	NA	NA	1
Huang et al. [62]	Random	NA	NA	NA	NA	NA	NA	2
Liu et al. [63]	Random	NA	NA	NA	NA	NA	NA	1
